# Starch Biocryogel for Removal of Methylene Blue by Batch Adsorption

**DOI:** 10.3390/polym14245543

**Published:** 2022-12-18

**Authors:** Tarawee Taweekarn, Worawit Wongniramaikul, Chanita Boonkanon, Chonthicha Phanrit, Wilasinee Sriprom, Wadcharawadee Limsakul, Wanchitra Towanlong, Chanadda Phawachalotorn, Aree Choodum

**Affiliations:** 1 Integrated Science and Technology Research Center, Faculty of Technology and Environment, Prince of Songkla University, Phuket Campus, Kathu, Phuket 83120, Thailand; 2 King Mongkut’s Institute of Technology Ladkrabang, Prince of Chumphon Campus, Chumphon 86160, Thailand

**Keywords:** dye removal, methylene blue, starch cryogel, batch adsorption

## Abstract

A green monolithic starch cryogel was prepared and applied for the removal of methylene blue (MB) using a batch system. The influence of various experimental parameters on MB adsorption was investigated. High removal efficiency (81.58 ± 0.59%) and adsorption capacity (34.84 mg g^−1^) were achieved. The Langmuir model better fitted the experimental data (determination coefficient (*R*^2^) = 0.9838) than the Freundlich one (*R*^2^ = 0.8542), while the kinetics of MB adsorption on the cryogel followed a pseudo-second-order model. The adsorption process was spontaneous and endothermic with an activation energy of 37.8 kJ mol^−1^ that indicated physical adsorption. The starch cryogel was used for MB removal from a wastewater sample collected from a local Batik production community enterprise in Phuket, Thailand, and a removal efficiency of 75.6% was achieved, indicating that it has a high potential as a green adsorbent for MB removal.

## 1. Introduction

Dyes are chemicals that can bind and impart color to different materials. Thus, they are used in many industries, including textiles, pharmaceutics, painting, leather, clothing, and printing [1,2,3]. Since their global production and consumption have increased, the dye business is estimated to grow annually by about 2–3% [1]. Note that ~2% of the dyes produced annually are discharged in effluents from manufacturing operations [4], which can cause serious water pollution due to their nonbiodegradable, highly toxic, carcinogenic, and mutagenic nature [2].

Methylene blue (MB), a thiazine cationic compound, is one of the most used dyes, especially for coloring and biological staining in many fields [1]. However, although MB can be utilized also for medical and pharmaceutical applications [1], it can cause several issues to human health, such as increased heart rate, vomiting, shock, cyanosis, jaundice, quadriplegia, and tissue necrosis [5,6]. Moreover, the discharge of MB effluents without proper treatment into natural streams has generated many environmental problems by reducing solar light penetration and hindering the photosynthetic activity of aquatic plants [7,8]. Therefore, highly effective and low-cost materials/methods should be developed to remove MB from wastewater

The major challenge in the removal of dyes lies in their complex aromatic molecular structures, which make them resistant to photodegradation, biodegradation, and oxidizing agents [9]. Thus, dye removal by traditional wastewater treatment technologies, e.g., physicochemical and biological processes, is difficult [1,3,10]. These methods also have other drawbacks: high costs, high energy consumption, sludge production, and abundant toxic byproducts [3,10]. Therefore, many researchers focused on the adsorption method due to its high efficiency, relatively facile operation, and low costs, as well as the abundance and easy recycling of adsorbent materials [1,3,10]. Various adsorbents have been tested for MB removal, including activated carbon [11,12], hydrogels [2], and biosorbents such as banana, orange, and pineapple peels [13], wheat shells [7], fava bean peel waste [1], and grape leaves [3]. Several studies have also been investigated to modify and convert conventional textile fibers to be adsorbents for dye removal, e.g., polypropylene [14,15], synthetic fiber [16], and chitosan polymer-coated cotton fibers [17]. Among them, cryogels derived from natural polymers, such as hydroxypropyl methylcellulose and bacterial cellulose nanocrystals [18] and alginate quasi-cryogels combined with clay [6,19] or graphene oxide [20], have attracted great interest because of their low environmental impact, fast response, and high efficiency.

In this study, a monolithic cryogel derived from starch was used as a green and low-cost adsorbent for MB removal. The costs and environmental impact were reduced by replacing commonly used synthetic polymers, such as polyvinyl alcohol [21], and toxic, expensive cross-linkers, such as glutaraldehyde [21,22,23,24], with, respectively, a natural polymer (starch) and limewater, achieving great removal efficiency. The effects of contact time, adsorbent dose, initial dye concentration, pH, and temperature on MB adsorption were investigated in a batch system. The adsorption mechanism, kinetics, and thermodynamic parameters were estimated to understand the nature of MB adsorption on the cryogel surface. Batch adsorption was selected as it is one of the most practical approaches used to adsorb pollutants from the liquid solution for the purification of water samples [25] with only a small amount of material required and less time consumption [26]. The study of adsorption by equilibrium in batch mode could provide critical information about the efficacy of a particular adsorbate-adsorbent system, enabling the prediction performance of the adsorbent before application on a larger scale [26].

## 2. Materials and Methods

### 2.1. Materials

MB was supplied by Merck (Darmstadt, Germany). The cryogel was synthesized from natural precursors consisting of rice flour (Erawan Brand, Nakhon Pathom, Thailand), tapioca starch (Jaydee Brand, Nakhon Pathom, Thailand), and limewater prepared from food-grade red lime purchased at a local supermarket in Kathu (Phuket, Thailand). Ethanol (95%, commercial grade) was supplied by High Science Co., Ltd. (Songkhla, Thailand). Ultrapure water from a water purification system (Merck, Darmstadt, Germany) was used for preparing all standard solutions.

### 2.2. Cryogel Preparation and Characterization

The starch cryogel was prepared as reported in our previous works [22,27,28,29]. Rice flour (12.5 g) and tapioca starch (3.75 g) were dispersed in limewater (130 mL) and heated under stirring for gelatinization until obtaining a clear and viscous solution. This solution was then cooled down at room temperature before being transferred (80 g) into a plastic syringe (50 mL, diameter of 3 cm and length of 10 cm) and frozen overnight at −20 °C. The resulting product was then thawed at room temperature for 3 h and underwent 3 freeze/thaw cycles. The obtained cryogel was removed from its container and cut into 1 cm lengths. The cryogel was soaked in 95% ethanol for 24 h, dried in an oven at 100 °C until achieving stable weight, and then stored in zip-lock plastic bags placed in a desiccator before usage.

The topography, before and after MB adsorption, and elemental composition of the cryogel were investigated using a field emission scanning electron microscopy (FESEM) system equipped with an energy-dispersive X-ray spectroscopy (EDX) detector (FEI, Eindhoven, The Netherlands). A Fourier-transform infrared spectroscopy (FTIR) instrument (Bruker, Bremen, Germany) was used to investigate the functional groups by the KBr technique.

### 2.3. Batch Adsorption and Determination of MB Content

Several experiments were conducted to investigate the adsorption characteristics of MB on the prepared cryogel, including the effects of adsorbent dose (1–6 pieces, 0.5–3.0 g), initial MB concentration (2.5–100 mg L^−1^), contact time (0–24 h), pH (4–9), and temperature (298, 308, and 318 K). As initial conditions determined with a preliminary study, one piece of cryogel (0.5 g) was placed into MB aqueous solutions (300 mL, 25 mg L^−1^) and magnetically stirred for 1 h at 298 K. The MB content was measured for three replicates using a spectrophotometer and a reported linear calibration curve at 664 nm [6] (1–25 mg L^−1^, y = (0.11x ± 0.01) + (0.22 ± 0.08), with determination coefficient (*R*^2^) = 0.9912), which was single-point recalibrated daily. The MB removal percentage was calculated as follows:

(1) $$\%\mathrm{Removal}=\frac{C_{i}{-C}_{e}}{C_{i}}\;\times \;100$$

where C_i_ (mg L^−1^) and C_e_ (mg L^−1^) are the initial and equilibrium concentrations of MB, respectively [1,29].

The amount of adsorbed MB at time t (q_t_: mg g^−1^) was estimated as the difference between C_i_ [30] and the MB concentration at t (C_t_: mg L^−1^) as follows:

(2) $$q_{t}=\frac{{(C}_{i}{-C}_{t})}{W}\times V$$

where V is the solution volume (L) and W is the dry mass of the adsorbents (g) [1,29].

### 2.4. Adsorption Isotherm

The MB adsorption isotherms on the proposed cryogel were investigated to understand the adsorption mechanism by varying C_i_ in the range of 5–50 mg L^−1^ while keeping constant the other conditions at their optimum values. The isotherms were constructed by plotting the adsorbed MB per mass unit of the adsorbent at equilibrium (q_e_, mg g^−1^) versus C_e_, followed by fitting with typically used Langmuir and Freundlich models. Both nonlinear and linear fitting methods were applied, and the goodness of the equilibrium model fitting was considered in terms of the sum of squared errors (SSE), residual standard deviation (RSD), and *R*^2^ [29].

The Langmuir isotherm equation can be expressed as [1,10,29,31]

(3) $$q_{e}=\frac{q_{\max}k_{L}C_{e}}{{1+k}_{L}C_{e}}$$

and

(4) $$\frac{1}{q_{e}}=\frac{1}{k_{L}q_{\max}}(\frac{1}{C_{e}})+\frac{1}{q_{\max}}$$

where q_max_ is the MB amount per mass unit of adsorbent at complete monolayer coverage (mg g^−1^) and k_L_ is the Langmuir constant relating to the adsorption strength (L mg^−1^); q_max_ and k_L_ can be derived from the slope and intercept of the linear plot between 1/q_e_ and 1/C_e_, respectively. The Langmuir isotherm was also investigated based on a dimensionless constant, known as the equilibrium parameter (R_L_), as follows: [29].

(5) $$R_{L}=\frac{1}{1+k_{L}C_{i}}$$

The Freundlich isotherm was also applied to the experimental data as follows:

(6) $$q_{e}{=k}_{F}C_{e}{}^{\frac{1}{n}}$$

and

(7) $${\mathrm{logq}}_{e}{=\mathrm{logk}}_{F}+\frac{1}{n}{\mathrm{logC}}_{e}$$

where k_F_ and n are the Freundlich constants related to adsorption capacity and intensity, respectively. The k_F_ and 1/n values were derived from the intercept and slope of the linear regressions fitted to log q_e_ versus log C_e_.

### 2.5. Adsorption Kinetics

Three models were used to investigate the adsorption kinetics of MB on the proposed cryogel: the Lagergren pseudo-first-order model [32,33], a pseudo-second-order model [32,34], and an intraparticle diffusion [29,32], 2011. The MB adsorption on cryogel was studied at different contact times (0−24 h), and the adsorption data were fitted with each model.

The Lagergren pseudo-first-order model was expressed as

(8) $$\frac{\mathrm{dqt}}{\mathrm{dt}}{=k}_{1}{(q}_{e}{-\;q}_{t})$$

which can be integrated for the boundary conditions t = 0, q_t_ = 0 and t = t, q_t_ = q_t_ to get

(9) $${\;\ln(q}_{e}{-\;q}_{t}{)=\mathrm{lnq}}_{e}{-k}_{1}t$$

where k_1_ is the pseudo-first-order kinetic rate constant (min^−1^) derived from the slope of the linear plot between $\ln\left(q_{e}-{\;q}_{t}\right)$ and t.

The pseudo-second-order model was expressed as

(10) $$\frac{\mathrm{dqt}}{\mathrm{dt}}{=k}_{2}{{(q}_{e}{-\;q}_{t})}^{2}$$

and

(11) $$\frac{t}{q}=\frac{1}{k_{2}q_{e}^{2}}+\frac{1}{q_{e}}t$$

where k_2_ is the second-order kinetic rate constant (min^−1^) derived from the slope of the linear plot between t/q_t_ and t.

The intraparticle diffusion model was expressed as [29,32].

(12) $$q_{t}{=k}_{p}t^{1/2}+c$$

where k_p_ is the intraparticle diffusion rate constant (mg g^−1^ min^−1/2^) derived from the slope of the plot between q_t_ and t^1/2^, and c is the intercept related to the thickness of the boundary layer. Intraparticle diffusion is the only rate-controlling step if the plot trendline is linear and passes through the origin.

### 2.6. Adsorption Thermodynamics

The adsorption of MB (100 mg L^−1^) on cryogel was investigated at 298, 303, and 318 K to observe the changes in thermodynamic factors, including Gibbs free energy (ΔG°), enthalpy (ΔH°), and entropy (ΔS°). ΔG° can explain the spontaneity and feasibility of an adsorption process, ΔH° informs about its nature (exothermic or endothermic), and ΔS° indicates the degrees of freedom in the system or the extent of molecular order/disorder [29]. ΔG° can be determined with the classical Van’t Hoff equation at any temperature (K) as follows:

(13) $$\Delta G^{0}{=-\mathrm{RTlnk}}_{d}$$

where R is the gas constant (8.314 J mol^−1^ K^−1^) [35] and k_d_ is the thermodynamic equilibrium constant (L g^−1^), which can be expressed as

(14) $$k_{d}=\frac{C_{a}}{C_{e}}$$

where C_a_ is the concentration of MB adsorbed on the cryogel at saturation (mg L^−1^).

Since ΔH° and ΔS° are linked to ΔG° as

(15) $$\Delta G^{0}=\Delta H^{0}-{T\Delta S}^{0}$$

they can be derived from the slope and intercept as follows:

(16) $${\mathrm{lnk}}_{d}=\frac{\Delta S^{0}}{R}-\frac{\Delta H^{0}}{R}(\frac{1}{T})$$

### 2.7. Real Sample Application

A real water sample was collected from the wastewater of local small and medium sized enterprises producing Batik clothing in Thalang, Phuket. It has a blue color (pH 6.23) with a maximum adsorption wavelength of 664 nm corresponding to the MB standard solution at a concentration of 3.85 mg L^−1^. Three pieces of cryogel (1.5 g) were placed into a 300 mL sample and magnetically stirred for 45 min at 298 K for batch adsorption.

## 3. Results

### 3.1. Cryogel Characterization

**Figure 1:**
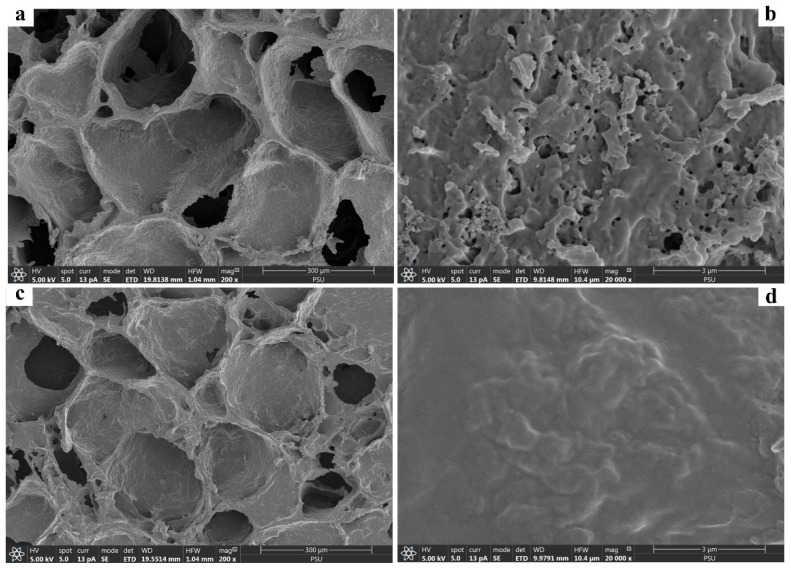
Field emission scanning electron micrographs of starch cryogel (**a**,**b**) before and (**c**,**d**) after adsorption of methylene blue.

The FESEM observation of the prepared starch cryogel (Figure 1) revealed a surface morphology containing macropores with an interconnected polymer network, consistent with our previous works [22,27,28,29] and cryogels prepared from synthetic polymers [36]. Micropores were also observed on the material wall (Figure 1b), providing many active sites for MB adsorption. Moreover, the cryogel surface seemed homogeneous due to its in-situ preparation without any composite with other materials. The formation of macroporous structure in polymeric cryogel is thus based on ice crystals without any implementation of other additives or filler [37]. It may also be attributed to the absence of microphase separation occurring during the dry cryogel preparation for the FESEM observation the same as reported in other hydrogels [38,39]. After adsorption, the observed pores were filled by MB molecules (Figure 1c,d). Thus, the cryogel surface became smoother and saturated, similar to what is reported for other materials [40,41].

**Figure 2:**
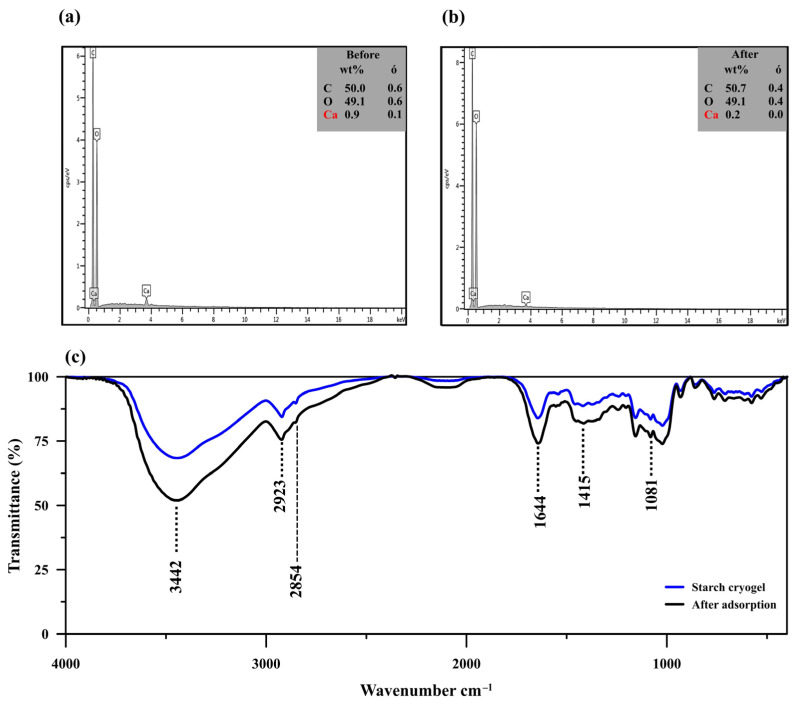
(**a**,**b**) Energy-dispersive X-ray spectra of starch cryogel before (**a**) and after methylene blue (MB) adsorption (**b**). (**c**) Fourier-transform infrared spectra of starch cryogel before and after MB adsorption.

The EDX analysis (Figure 2a,b) showed a decrease in the calcium content from 0.9 to 0.2 wt% after MB adsorption, along with a slight reduction in the carbon content (from 50.0 to 49.1 wt%) and an increase in the oxygen one (from 49.1 to 50.7 wt%). Since limewater was used as the cross–linker for cryogel preparation, excess calcium ions might have been trapped within the material and consequently released into the aqueous solution during the adsorption process, reducing the calcium content after MB adsorption. The high content of carbon in the prepared cryogel (~50.0%) allowed efficient adsorption due to its porous structure, similar to carbonaceous materials [42].

The FTIR spectrum of the cryogel showed an absorption band at 3443 cm^−1^ attributed to O–H stretching from the starch molecules interacting with the calcium ions in their surroundings (Figure 2c). MB adsorption leads to the increase of signal intensity at 3442 cm^−1^ due to the overlapping with –NH/–OH of MB [43]. Another peak was observed at 2921 and 2923 cm^−1^ before and after MB adsorption, respectively; it was assigned to the C–H stretching in the starch molecules and is also commonly observed in MB spectra at 2924 cm^−1^ [43]. The absorption band at 1645 and 1641 cm^−1^ before and after MB adsorption, respectively, was attributed to H–O–H bending from water molecules and/or C–O bending in amylopectin [44,45,46]. This peak was more intense after MB adsorption since it likely overlapped with the N–H bending vibration generally detected at 1599 cm^−1^ [42]. The peak observed at 1419 cm^−1^ and those in the 1081–931 cm^−1^ range were assigned to C–H symmetrical scissoring of the CH_2_OH moiety and C–O bonding in amylopectin of the starch molecules [44,45,46]. The spectrum after MB adsorption revealed remarkable intensity changes with little wavenumber variation (<10 cm^−1^), which indicates that the MB adsorption mechanism may consist of physical interactions, such as hydrogen bonding, electrostatic force, van der Waals force, or hydrophobic interactions [40,47].

### 3.2. Batch System

#### 3.2.1. Adsorbent Dose

**Figure 3:**
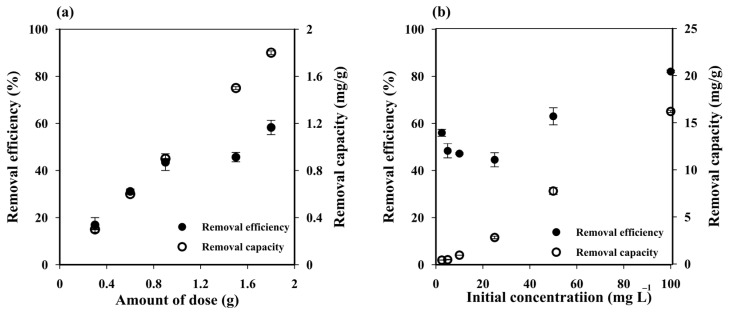
Influence of (**a**) cryogel dose and (**b**) initial methylene blue (MB) concentration on the MB removal efficiency and capacity of starch cryogel.

Increasing the cryogel dose from one to six pieces (0.5 to 3 g) enhanced the MB removal efficiency from 16.90 ± 3.11% to 58.27 ± 3.03% (Figure 3a) due to the increased number of active sites of sorbent [48]. The adsorbent dose for three pieces (~1.5 g) was selected as the optimum condition.

#### 3.2.2. Influence of Initial MB Concentration

The MB removal capacity of the prepared cryogel increased along with Ci (Figure 3b), suggesting that the initial MB concentration is an important factor in the adsorption process. The low removal capacity at low C_i_ (0.28 ± 0.01 mg g^−1^ at 2.50 mg L^−1^) was attributed to the unsaturation of the cryogel adsorption sites, which would increase until reaching a critical point with no more significant changes in removal capacity due to a saturation MB concentration on them. These results suggest that the maximum adsorption capacity of the prepared cryogel can be achieved at C_i_ > 100 mg L^−1^, where the removal efficiency reached 81.58 ± 0.59%. The removal efficiency slightly decreased with increasing MB concentrations from 2.50 to 25 mg L^−1^, but then increased at higher initial concentrations. Since the initial dye concentration provides the driving force to overcome the resistance to the mass transfer of dye between the aqueous and the solid phase, thus the higher the initial concentration, the higher the driving force, and thus the higher amount of dye that can be adsorbed while saturation takes place very fast [49]. At a short contact time, e.g., 45 min used in this experiment, the lower amount of dye was expected to be absorbed on the adsorbent at lower initial concentration leading to a lower removal efficiency. A small decrease in removal efficiency with increasing MB concentrations from 2.50 to 25 mg L^−1^ may attributed to the competition of dye to attach on vacant adsorbent sites at the range of concentrations with 45 min contact time [49].

#### 3.2.3. Influence of pH

**Figure 4:**
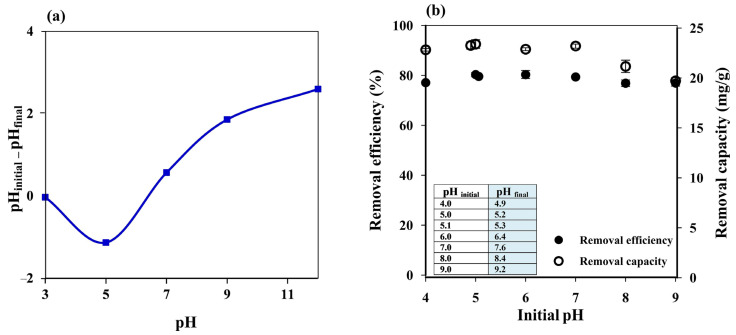
(**a**) Point of zero charge of starch cryogel. (**b**) influence of initial pH on the MB removal efficiency and capacity of starch cryogel.

The pH of a dye solution is an essential factor that can influence the adsorption process since it may affect the charge on the adsorbent surface. The point of zero charge (pHpzc) of the prepared cryogel was thus investigated as reported in the literature [29,50]. The cryogel showed the pH_pzc_ at pH ~6.4 (Figure 4a), which means that the cryogel surface will be positively charged at pH < 6.4 and negatively charged at pH > 6.4.

Since MB is a cationic dye with pKa 3.8 [51] and the cryogel pH_pzc_ was 6.4, high adsorption efficiency was expected at an MB solution pH above 6.4 due to electrostatic interactions between the positively charged MB and the negatively charged cryogel. Increasing the pH of the MB solution from 4 to 5 improved the removal efficiency from 77.08 ± 1.02% to 80.31 ± 0.84%, which then remained constant from pH 5.1 (without pH adjustment) to pH 7 (Figure 4b); in the same pH range (4–7), the removal capacity increased from 22.82 ± 0.13 to 23.39 ± 0.44 mg g^−1^. The removal efficiency remained constant, while the removal capacity decreased at pH 8 (21.16 ± 0.62 mg g^−1^ and 76.86 ± 1.33%, respectively) and pH 9 (19.71 ± 0.31 mg g^−1^ and 76.86 ± 1.20%, respectively), although the adsorbent could still be considered highly efficient. Since the cryogel was prepared by ionic cross-linking of starch with limewater, Ca^2+^ and/or OH^−^ ions could be released into the solution during the adsorption process, affecting the solution pH. Thus, the final pH of the MB solution (pH_final_), i.e., after the adsorption process (insert of Figure 4b), was also considered.

The lower adsorption efficiency observed at pH 4 (pH_final_ = 4.9) may be attributed to the repulsion between the positively charged cryogel surface and positively charged MB. However, it can still be considered highly efficient (~77%) due to hydrogen bonding between cryogel and MB, which is a dominant adsorption mechanism under acidic conditions [42]. Hydrogen bonding might have still occurred at pH 5 (pH_final_ = 5.2) and 5.1 (pH_final_ = 5.3) with lower electrostatic repulsion since the cryogel pH_pzc_ was ~6.4, resulting in higher adsorption efficiency than at pH 4. At pH 6 and 7 (pH_final_ = 6.4 and 7.6), most of the cryogel surface should have been neutral and, thus, the constant adsorption efficiency may be due to hydrophobic–hydrophobic interactions. The reduction in the removal capacity at pH > 8 might have contributed to the charge screening effect of excess Na^+^ ions that prevented effective electrostatic attractions between CO– on the cryogel surface and positively charged MB [2,52], and the Ca^2+^ ions released from the cryogel might have supported this screening effect.

Therefore, the adsorption experiments were performed using the MB solution without pH adjustment (pH 5.1) to save the cost of chemicals required for such a treatment in real applications. Hydrogen bonding between MB and the cryogel surface is thus proposed as the dominant adsorption mechanism, which agrees well with the FTIR results where N–H bending at 1599 cm^−1^ [42] may overlap with H–O–H bending from water molecules and/or C–O bending in amylopectin at 1641 cm^−1^ [44,45,46], resulting in a more intense peak after MB adsorption.

#### 3.2.4. Influence of Contact Time

**Figure 5:**
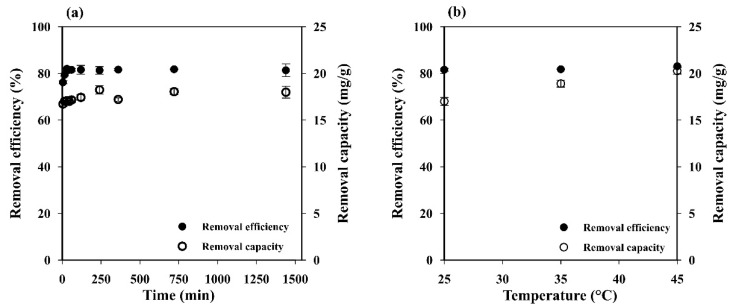
Influence of (**a**) contact time and (**b**) temperature on the MB removal efficiency and capacity of starch cryogel.

Figure 5a illustrates the effect of contact time on adsorption capacity and removal efficiency. The MB adsorption increased rapidly up to 76.19 ± 0.66% within the first 5 min and reached the equilibrium (81.96 ± 0.08%) within 30 min. This behavior may be attributed to the porous network structure with high permeability and easily accessible adsorption sites of the starch cryogel, which could accelerate the intraparticle diffusion of MB on its surface [2]. The adsorption capacity also increased during the time from 16.73 ± 0.28 mg g^−1^ at 5 min to 22.51 ± 0.44 mg g^−1^ at 12 h and remained constant afterward. This indicates that the MB adsorption capacity of the prepared cryogel mainly depends on its specific surface area.

#### 3.2.5. Influence of Temperature

Since temperature is an important parameter for controlling the strength of adsorptive forces between adsorbent and adsorbate molecules, its influence was investigated at 298, 308, and 318 K. The adsorption capacity increased a little (from 17.08 ± 0.23 to 18.43 ± 0.51 mg g^−1^) when increasing the temperature (Figure 5b). Adsorption processes are generally exothermic, i.e., the adsorption capacity decreases with increases in the temperature. However, some of them are endothermic, i.e., the adsorption capacity increases along with the temperature because the adsorbate molecules need the energy to move around and penetrate deeper into the adsorbent at increasing temperatures [42]. Thus, the results presented here indicate that the MB adsorption on the prepared cryogel is an endothermic process where the MB molecules need the energy to move around and penetrate deeper into its wall micropores at increasing temperatures [42], and this was most likely due to physical rather than chemical adsorption [35].

### 3.3. Adsorption Isotherm

**Figure 6:**
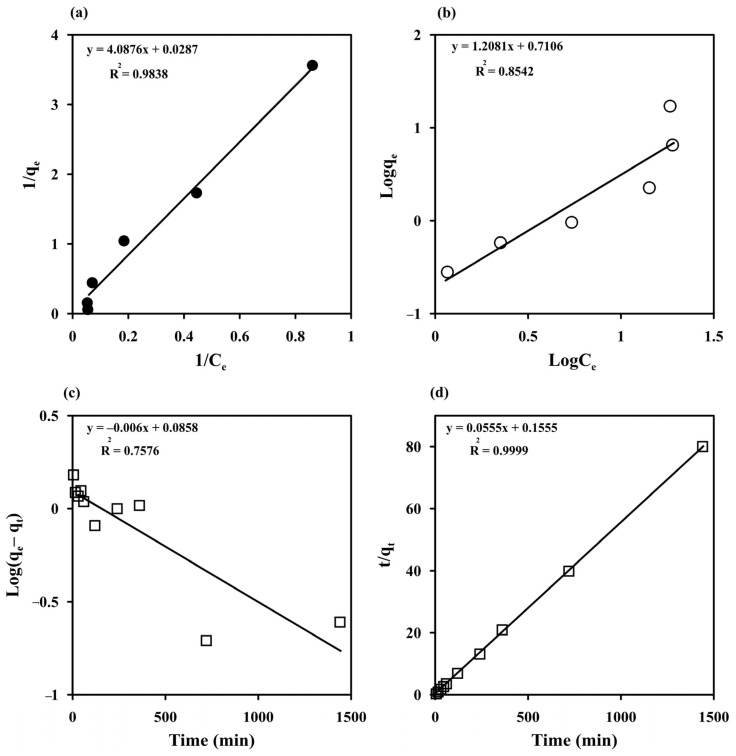
Adsorption isotherm of methylene blue (MB) on starch cryogel (**a**) the Langmuir and (**b**) Freundlich models and its adsorption kinetics (**c**) the Lagergren pseudo-first-order and (**b**) the pseudo-second-order models.

The Langmuir and Freundlich models were used to fit the experimental data obtained by varying C_i_ to study the MB adsorption behavior of the prepared cryogels (Figure 6a,b). The Langmuir and Freundlich parameters, SSE, SD, and *R*^2^ estimated with both nonlinear and linear fitting methods are listed in Table 1. The experimental results were better fitted with the Langmuir model in linearized forms than with the Freundlich model with the higher *R*^2^ (0.9838) and lower SSE (0.14). This indicates a homogeneous surface for monolayer adsorption of MB, in agreement with the FESEM results and previous reports on MB adsorption by other materials, such as a magnetic graphene oxide/carbon nanotube composite (q_max_ = 24.88 mg g^−1^ for magnetic graphene oxide) [8], magnetic Fe_3_O_4_/C core–shell nanoparticles (q_max_ = 31.18 mg g^−1^) [53], cross-linked porous starch (q_max_ = 9.46 mg g^−1^) [54], and grape leave waste (q_max_ = 0.2 mg g^−1^) [3], but with a higher maximum adsorption capacity of 34.84 mg g^−1^. The R_L_ values ranged from 0.08 to 0.77, indicating a favorable adsorption process, which confirms that the Langmuir model is suitable for describing MB adsorption on monolithic starch cryogel.

### 3.4. Adsorption Kinetics

The experimental data obtained from varying the contact time from 5 to 1440 min at different temperatures (298–313 K) were fitted with the Lagergren pseudo-first-order model [32,33] and the pseudo-second-order model [32,34] to understand the sorption kinetics (Figure 6c,d). The high degree of linearity (*R*^2^ = 0.9999) obtained from the pseudo–second–order model demonstrated its ability to describe the kinetics of MB adsorption, similar to previous reports [2,3,8,54]. Furthermore, the adsorption capacity measured at 298 K (q_e exp_ = 17.08 mg g^−1^) was close to the equilibrium adsorption capacity calculated with the pseudo–second–order model (q_e cal_ = 18.02 mg g^−1^) (Table 2). The adsorption kinetics of MB on the starch cryogel surface can therefore be best described by the pseudo-second-order model. The k_2_ value increased from 0.02 to 0.05 g mg^−1^ min^−1^ with increasing the temperature, indicating that a higher temperature enhances the adsorption with an endothermic process. The estimated k_2_ value for starch cryogel (0.0005 g mg^−1^ h^−1^) was lower than for epichlorohydrin cross-linked porous starch (0.0035–0.0093 g mg^−1^ h^−1^) [54], possibly due to the higher surface area of cross-linked porous starch granules. The half-life of adsorption (t_1/2_), i.e., the time needed to adsorb 50% of MB at the equilibrium, was estimated as [29,35].

(17) $$t_{1/2}=\frac{1}{k_{2}q_{e}}$$

It decreased from 2.88 min at 298 K to 1.15 min at 318 K, indicating faster adsorption at higher temperatures, which might be attributed to the increased mobility and mass transfer rate of MB towards the cryogel surface and its internal pores.

Since the adsorption process may involve intraparticle diffusion, the intraparticle diffusion model [29,32] was also investigated to better understand the adsorption kinetics of MB on the prepared cryogel. The q_t_ vs. t^1/2^ plot was not linear (*R*^2^ = 0.6033), suggesting that intraparticle diffusion does not play an important role in the adsorption process of MB on the starch cryogel. Moreover, the intercept did not pass through the origin, indicating that intraparticle diffusion is not the only rate-controlling step.

### 3.5. Adsorption Thermodynamics

The activation energy (E_a_: kJ mol^−1^) of MB adsorption on the prepared cryogel could be estimated as

(18) $${\mathrm{lnk}}_{2}={\;\mathrm{lnk}}_{0}\;–\frac{\mathrm{Ea}}{\mathrm{RT}}$$

where k_0_ is a factor independent of temperature. In general, the sorption process is classified as physical adsorption when E_a_ < 40 kJ mol^−1^ [29,35], film-diffusion controlled if E_a_ < 16 kJ mol^−1^, particle-diffusion controlled if E_a_ is 16–40 kJ mol^−1^ [55], and chemical adsorption when E_a_ > 40 kJ mol^−1^ [29,35]. Here, the estimated Ea value of 37.8 kJ mol^−1^ indicates the physical adsorption of MB on the prepared cryogel with particle diffusion control, although the value is very close to chemical adsorption.

The ΔG°, ΔH°, and ΔS° values derived from the experimental data are summarized in Table 3. The positive ΔS° value of 23.14 J mol^−1^ K^−1^ suggests the disorder degree and an increase in particle randomness at the cryogel/liquid interface during the adsorption process [29,35]. The negative ΔG° values of −3.72, −3.96, and −4.19 kJ mol^−1^ obtained at 298, 308, and 318 K, respectively, indicate that MB adsorption on the prepared cryogel is spontaneous and more favorable at higher temperatures. Moreover, the small values of ΔG° in the range from −20 to 0 kJ mol^−1^ suggest a physical adsorption process, similar to what is reported for epichlorohydrin cross-linked porous starch [54]. The positive ΔH° values indicate physisorption and endothermic process. These results agree well with the FTIR results and the observed pH, confirming physical adsorption as the dominant mechanism of MB adsorption on the starch cryogel.

### 3.6. Real Sample Application

The monolithic cryogel was used for MB removal from a wastewater collected from the local Batik production community enterprise in Phuket, Thailand. The concentration of MB was reduced from 3.85 to 0.94 mg L*^−^*^1^ after batch adsorption, indicating a removal efficiency of 75.6%.

Therefore, the starch cryogel could be used as a green adsorbent for MB removal without the need for any active material and/or filler, although its removal capacity may be less than other composites (Table 4). The preparation of the cryogel is also simpler with fewer chemicals required and less waste generated, making it more environmentally friendly.

## 4. Conclusions

Starch cryogel can be used as a green adsorbent for MB removal with high efficiency in the batch system (removal efficiency > 80% in synthetic MB solution and >75% in real sample). The MB adsorption on the starch cryogel was dependent on pH, initial dye concentration, contact time, and temperature. The adsorption experiments can be performed without pH adjustment (pH 5.1) at ambient temperature to save the cost of chemicals and electricity required for such a treatment in real applications, while MB adsorption can reach the equilibrium within 45 min due to the porous network structure with high permeability and easily accessible adsorption sites of the starch cryogel. The experimental data were better fitted with the Langmuir model (*R*^2^ = 0.9838) indicating a homogeneous surface for monolayer adsorption of MB, while the kinetics of MB adsorption on the cryogel followed a pseudo-second-order model. The adsorption process was spontaneous and endothermic with an activation energy of 37.8 kJ mol^−1^, indicating a physical process. A starch cryogel can be used for MB removal without the need for any active material and/or filler, although its removal capacity (adsorption capacity 34.84 mg g^−1^) may be less than other composites. The preparation of the cryogel is also simpler with fewer chemicals required and less waste generated, making it more environmentally friendly.

## Figures and Tables

**Table 1:** Parameters derived from applying the Langmuir and Freundlich models to the batch adsorption data.

Model	Parameter	Linear	Non-Linear
Langmuir	q_m_ (mg g^−1^)	34.84	370,735
	K_L_ (L mg^−1^)	0.12	0.000001
	*R* ^2^	0.9838	0.6041
	SD	0.19	4.96
	SSE	0.14	98.30
Freundlich	K_F_ (mg^1−n^L^n^g^−1^)	0.20	0.000002
	1/n	1.21	5.27
	*R* ^2^	0.8542	0.6327
	SD	0.20	4.43
	SSE	0.15	78.54

**Table 2:** Parameters derived from applying the Lagergren pseudo-first-order model and the pseudo-second-order model to the batch adsorption data.

Model	Parameter	Temperature (K)		
		298	308	318
Pseudo-first-order	q_e_ (mg g^−1^)	1.22	1.24	2.88
	k_1_ (min^−1^)	0.0014	0.0046	0.0271
	*R^2^*	0.7576	0.6224	0.8054
Pseudo-second-order	q_e_ (mg g^−1^)	18.02	18.28	18.48
	k_2_ (g mg^−1^ min^−1^)	0.0193	0.0296	0.0472
	*R^2^*	0.9999	0.9999	0.9999

**Table 3:** Thermodynamic parameters for adsorption of MB on starch cryogel derived from the batch adsorption data.

**ΔH° (kJ/mol)**	**ΔS° (J/(mol.K))**	**ΔG° (kJ/mol)**		
		**298 K**	**303 K**	**318 K**
3.17	23.14	−3.72	−3.96	−4.19

**Table 4:** Comparison of various parameters of starch cryogel and other cryogel materials for MB removal.

Parameter	[18]	[19]	[20]	[6]	This Work
Supporting material	Hydroxypropyl methylcellulose	Alginate	Alginate	Alginate	Starch
Composite	Bacterial cellulose nanocrystals	Montmorillonite	Graphene oxide	Montmorillonite	-
Chemical required	Bacterial cellulose CHCl_3_, H_2_SO_4_/HCl, HPMC, Citric acid, Sodium hypophosphite	Sodium alginate, Montmorillonite, CaCl_2_, NHS, MES, Cys, EDC	Sodium alginate, Graphene oxide, CaCl_2_	Sodium alginate, Montmorillonite, CaCl_2_	Rice flour, Tapioca starch, Limewater
Preparation	Complicate	Complicate	Easier	Easier	Easier
Adsorption system	Batch	Batch	Batch	Batch	Batch
q_e_ (mg/g)	-	559.7 g/g	122.26	181.8	34.84
Cost/g	most expensive	more expensive	expensive	expensive	cheap (0.003 USD)
Real sample	x	x	x	x	✓

HPMC: Hydroxypropyl methylcellulose; NHS: *N*-Hydroxysuccinimide; MES: 2-(*N*-Morpholino) ethane sulfonic acid hydrate; Cys: Cystamine hydrochloride; EDC: *N*-(3-Dimethylaminopropyl)-*N*′-ethyl carbodiimide hydrochloride.

## Data Availability

Not applicable.
